# Behavioral and physiological female responses to male sex ratio bias in a pond-breeding amphibian

**DOI:** 10.1186/1742-9994-9-24

**Published:** 2012-09-18

**Authors:** Kristine L Grayson, Stephen P De Lisle, Jerrah E Jackson, Samuel J Black, Erica J Crespi

**Affiliations:** 1 Department of Biology, University of Virginia, Charlottesville, VA, 22904, USA; 2 Department of Biology, Virginia Commonwealth University, Richmond, VA, 23284, USA; 3 Mountain Lake Biological Station, University of Virginia, Pembroke, VA, 24136, USA; 4 Department of Biology, Vassar College, Poughkeepsie, NY, 12604, USA; 5 School of Biological Sciences, Washington State University, Pullman, WA, USA

**Keywords:** Leukocytes, Male-biased sex ratio, Mating behavior, Microhabitat use, *Notophthalmus viridescens*, Red-spotted newt, Sexual conflict, Stress

## Abstract

**Introduction:**

The phenomenon of sexual conflict has been well documented, and in populations with biased operational sex ratios the consequences for the rarer sex can be severe. Females are typically a limited resource and males often evolve aggressive mating behaviors, which can improve individual fitness for the male while negatively impacting female condition and fitness. In response, females can adjust their behavior to minimize exposure to aggressive mating tactics or minimize the costs of mating harassment. While male-male competition is common in amphibian mating systems, little is known about the consequences or responses of females. The red-spotted newt (*Notophthalmus viridescens*) is a common pond-breeding amphibian with a complex, well-studied mating system where males aggressively court females. Breeding populations across much of its range have male-biased sex ratios and we predicted that female newts would have behavioral mechanisms to mitigate mating pressure from males. We conducted four experiments examining the costs and behavioral responses of female *N. viridescens* exposed to a male-biased environment.

**Results:**

In field enclosures, we found that female newts exposed to a male-biased environment during the five-month breeding season ended with lower body condition compared to those in a female-biased environment. Shorter-term exposure to a male-biased environment for five weeks caused a decrease in circulating total leukocyte and lymphocyte abundance in blood, which suggests females experienced physiological stress. In behavioral experiments, we found that females were more agitated in the presence of male chemical cues and females in a male-biased environment spent more time in refuge than those in a female-biased environment.

**Conclusions:**

Our results indicate that male-biased conditions can incur costs to females of decreased condition and potentially increased risk of infection. However, we found that females can also alter their behavior and microhabitat use under a male-biased sex ratio. Consistent with surveys showing reduced detection probabilities for females, our research suggests that females avoid male encounters using edge and substrate habitat. Our work illustrates the integrated suite of impacts that sexual conflict can have on the structure and ecology of a population.

## Introduction

The sex ratio of a population can play an important and complex role in the ecology of a species. A bias in the operational sex ratio (OSR) of a population, the ratio of reproductively active males to females, can lead to intense mate competition in the more common sex and therefore, increased sexual selection on traits that improve mating success [1]. These traits include behaviors that increase the number of mating opportunities and the likelihood of successful mating attempts [2,3]. Males often evolve coercive behaviors, such as forced copulations or harassment, which can decrease female fitness [4]. Male harassment can be a consequence of sexual conflicts over the optimal number of matings, which is predicted to be larger for males than for females [5], and can result in increased female acceptance of matings [6]. While the display of aggressive behaviors could have reproductive benefits for males, this increased competition among males for mates can have negative fitness consequences for females. Male harassment behavior has been shown to be costly to females in a variety of species (reviewed in [3,4]). In such cases, females are predicted to adopt resistance traits that reduce mating frequency or minimize costs of coercion [7].

Under male-biased sex ratios, harassment behaviors and negative impacts on female fitness can be intensified. For example, an excess of males results in higher mating rates in stream water striders (*Aquarius remigis*), both in field and experimental venues, which results in decreased activity and foraging time for female water striders [8]. In a viviparous lizard (*Lacerta vivipara*), a male-biased sex ratio can have a significant impact on immediate female condition and reduce lifetime reproductive output [9]. Sexual conflict can also have significant impacts on the sex ratio dynamics within a population over time and space [10] and affect the growth rate of a population [11].

**Figure 1:**
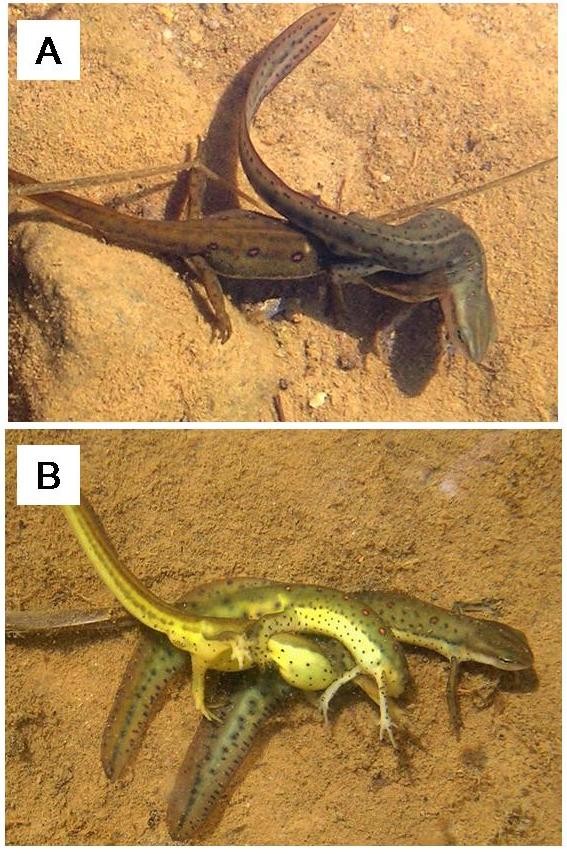
**Courtship posture of red-spotted newts,* N. viridescens*.** A single male captures and courts a female (**A**), while rival males can also attach and attempt to court the female or dislodge the male (**B**). This posture is termed amplexus. Photographs by K. Grayson.

In amphibians, male-male competition can be so intense in some species that females can drown due to harassment from competing males [12,13]; however, aside from these extreme cases, little is known about the fitness consequences of mate competition for female amphibians. Here, we examined the behavioral and physiological effects of a male-biased environment on female red-spotted newts (*Notophthalmus viridescens*) to better understand how females are affected by mate competition behaviors exhibited by males (Figure 1). This species is a common pond-breeding amphibian across the eastern United States and Canada with a highly competitive mating system consisting of complex courtship and mating behaviors (e.g., [14-16]). Aquatic breeding populations are often male-biased (e.g., [17-19]), likely due to differences in breeding frequency. At the study site, male newts usually breed every year and female newts often remain in the terrestrial habitat and skip breeding opportunities [20]. This results in a population of residents and migrants, where individuals can switch between the two strategies depending on their condition and reproductive state [21]. Thus, for the population of newts we studied, the sex ratio in the breeding pond represents the OSR due to non-reproductive individuals remaining in the forest. 

The majority of courtship and mating occurs at the study site from March to June [15,22]: male newts actively capture female newts and grasp the pectoral region using their hind limbs. This courtship position is termed amplexus, and in *N. viridescens* this behavior is used by males when female newts are initially unresponsive to courtship ([23]; Figure 1A). Males deposit hormonal secretions on the female skin during amplexus that have been shown to influence female receptivity to mating [24]. After amplexus, males disengage and deposit a spermatophore, which a female may accept by moving forward and picking it up in her cloaca or may reject by fleeing [15,23]. Amplexus encounters with females are highly competitive and often rival males also attach to the female ([22]; Figure 1B), which can displace the courting male or interfere with spermatophore transfer [14,15]. Courting males defend females against rivals by increasing the duration of amplexus [25,26]. Females can mate and accept spermatophores from multiple males, as well as store sperm over long periods [27]. Eggs are laid singly over several weeks and wrapped in aquatic vegetation [28,29].

Aggressive male behaviors likely have many fitness costs for females. Repeated harassment of females by males can stimulate a physiological stress response that can result in elevated metabolism and reductions in feeding, which can contribute to body weight loss [30]. Fitness costs could also manifest from stress-induced suppression of physiological function, such as the immune system, caused by elevations in glucocorticoids or by reductions in energetic stores (reviewed in [31]). Therefore, in addition to measuring changes in body condition, fitness costs can be assessed through measuring the activity of immune system as proxy of stress [32].

Faced with costs from male mating behaviors, females are predicted to evolve mechanisms to minimize the impacts of interactions with males. For example, studies in other species suggest that females can adopt counterstrategies to reduce the costs of male mating pressure or to reduce the frequency of matings. These measures could be changes in behavior, such as female bees that alter foraging behavior to avoid males [33], or changes in morphology, such as female water striders increasing abdominal spines [34]. Indeed, Rohr *et al.*[35] showed that *N. viridescens* can use chemical cues to assess operational sex ratio and mate competition. Although their experiments were focused on how male newts assess intrasexual competition, Rohr *et al.*[35] also noted that females avoided large groups of males and suggested that male groups could be costly to females. We suspected that female newts may be altering their behavior to avoid male mating attempts during mark-recapture pond surveys. During that study, the ability to capture pond-dwelling female newts in aquatic traps was much lower compared to males (estimated detection probability = 0.66 ± 0.03 for males and 0.18 ± 0.07 for females), but this difference was not seen in migrating newts captured by terrestrial pitfall traps [21].

We conducted this study to examine how female red-spotted newts respond to male mating behaviors and specifically, to test the hypothesis that female newts alter habitat use in response to a male-biased sex ratio. To test this hypothesis, we conducted a set of experiments to 1) determine if a male-biased environment has negative effects on long-term and short-term female condition in two separate field enclosure experiments, and 2) test if female *N. viridescens* change their behavior and microhabitat in response to male-biased environments.

## Results

### Body condition is negatively impacted by long-term exposure to a male-biased sex ratio

The long-term effect of a male-biased sex ratio on individual condition was tested over a five month period using large pond enclosures [36]. Survival was high with 93.1% of newts recovered alive after five months in field enclosures and was not significantly different based on treatment or newt sex. Of the 720 newts that began the experiment, 24–58% of male newts and 53–69% of female newts from each enclosure became migratory, leaving 180 male and 117 female resident newts in the enclosures at the end of the experiment (see [36] for detailed migration results). Briefly, density significantly influenced migration, with more newts leaving as density increased. Across the treatments, more female newts migrated compared to males. However, sex ratio treatment did not significantly affect migration outcome or timing, nor were any of the interactions between the main factors significant [36]. Thus, the proportion of males increased in all of the enclosures over the course of the experiment as individuals migrated out, but these changes in sex ratio were similar across the treatments. We used newts that remained in the enclosures until the end of the experiment (residents) to examine change in mass.

**Figure 2:**
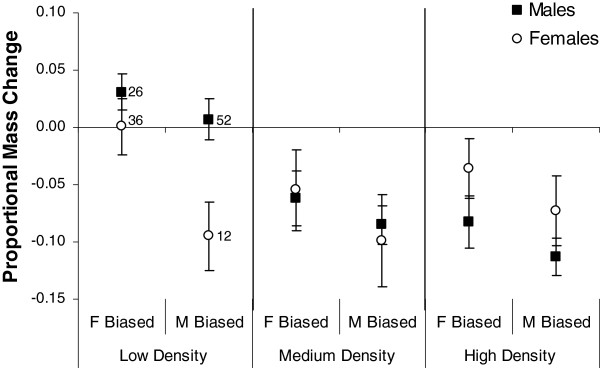
**Effect of long-term exposure to a male- or female-biased sex ratio on body condition.** Mean proportional change in mass per treatment of individual *N. viridescens* males and females. Only resident individuals, as opposed to individuals that migrated from enclosures, were used because residents 1) retained the aquatic phenotype and therefore changes in mass are solely due to changes in body condition and 2) are not confounded by differences in the amount of time exposed to the treatments. Means are ± 1 SE. The number of individuals is indicated for each point (total *N* = 180 male and 117 female newts).

Residents declined in mass for all treatments except for the low density/female-biased treatment for both sexes and the low density/male-biased treatment for males (Figure 2). Accordingly, density was found to have a significant effect on mass (*F*_2,53_ = 6.83, *P* = 0.002). Sex ratio treatment was also significant (*F*_1,53_ = 6.79, *P* = 0.01), with individuals losing 144% more mass in the male-biased treatment than in the female-biased treatment. This effect was consistent across the density treatments, as the interaction between the density treatment and sex ratio treatment was not significant (*F*_2,53_ = 0.27, *P* = 0.76). We also tested for differences between males and females in their mass response. The main effect of sex was not significant (*F*_1,53_ = 0.22, *P* = 0.64), but the interaction between density and sex was significant (*F*_2,53_ = 3.41, *P* = 0.04), likely due to the loss in mass seen in resident females in the low density/male-biased enclosures (Figure 2). The remaining interaction between sex and the sex ratio treatment and the three-way interaction were not significant (*F* < 0.95, *P* > 0.34).

### Short-term exposure to a male-biased sex ratio negatively impacts immune function, but not body condition

**Figure 3:**
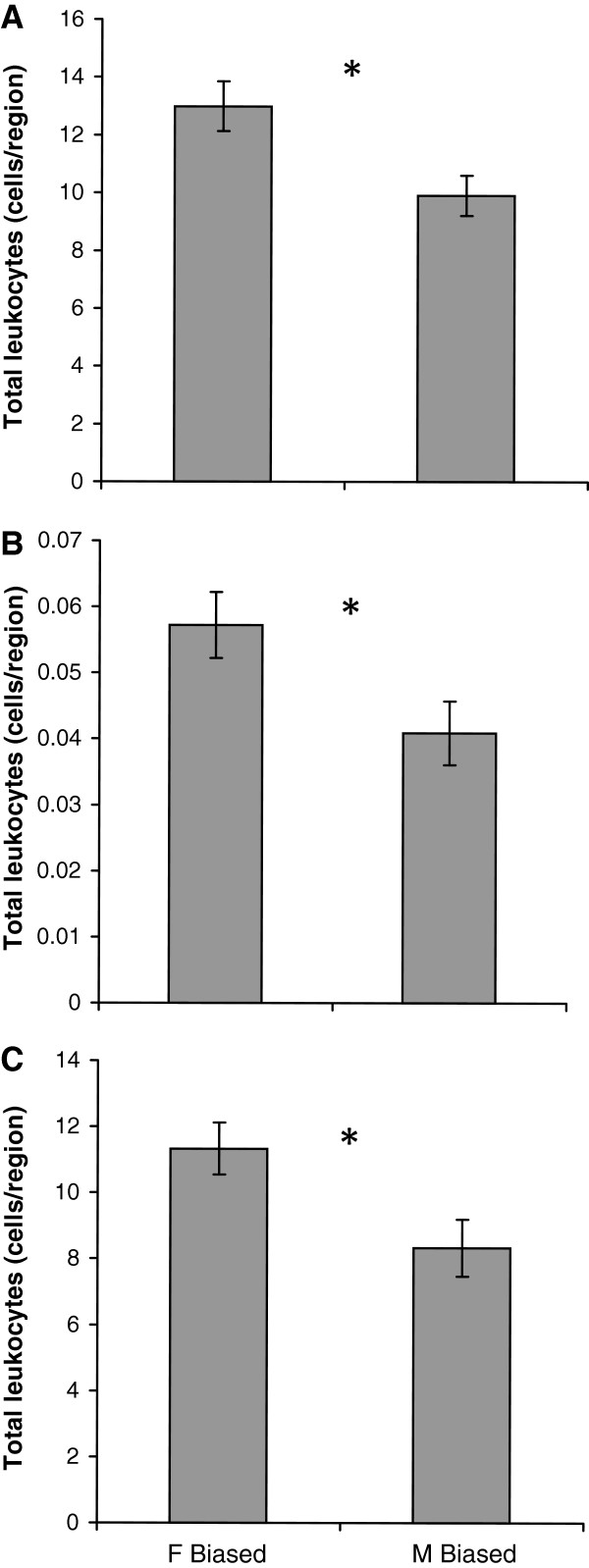
**Effect of short-term exposure to a male-biased sex ratio on immune function.** Mean ± 1 SE of **A**) total leukocyte abundance, and **B**) proportion of total leukocytes to total cells counted, and **C**) lymphocyte abundance in blood samples taken from *N. viridescens* females housed in female-biased enclosures (*N* = 12) or male-biased enclosures (*N* = 10) for five weeks. * indicates *P* < 0.05; raw means are shown here although these data were transformed for statistical analysis (see Methods).

We conducted a second field experiment to examine the short-term effects of a male-biased sex ratio on female condition over a five week period using smaller pond enclosures. All newts survived during the experiment. Five weeks of exposure to the sex ratio treatments did not affect the final mass (*t* = 0.63, *P* = 0.27) or change in mass in females (*t* = 0.36, *P* = 0.36). Abundance of circulating erythrocytes in females did not vary with treatment (*t* = −0.025, *P* = 0.980), but females that were subjected to a male-biased sex ratio exhibited significantly fewer circulating total leukocytes (*t =* 2.76*, P =* 0.012) by approximately 28% (Figure 3A), and the proportion of leukocytes:total cells also was lower (*t =* 2.39*, P =* 0.0286; Figure 3B). Specifically, lymphocyte abundance was reduced in females housed in male-biased containers (*t =* 2.61*, P =* 0.017; Figure 3C), although there were no significant differences between treatments in the abundance of any other type of white blood cells (*P >* 0.05).

### Female newts respond behaviorally to chemical cues from a male-biased sex ratio

**Figure 4:**
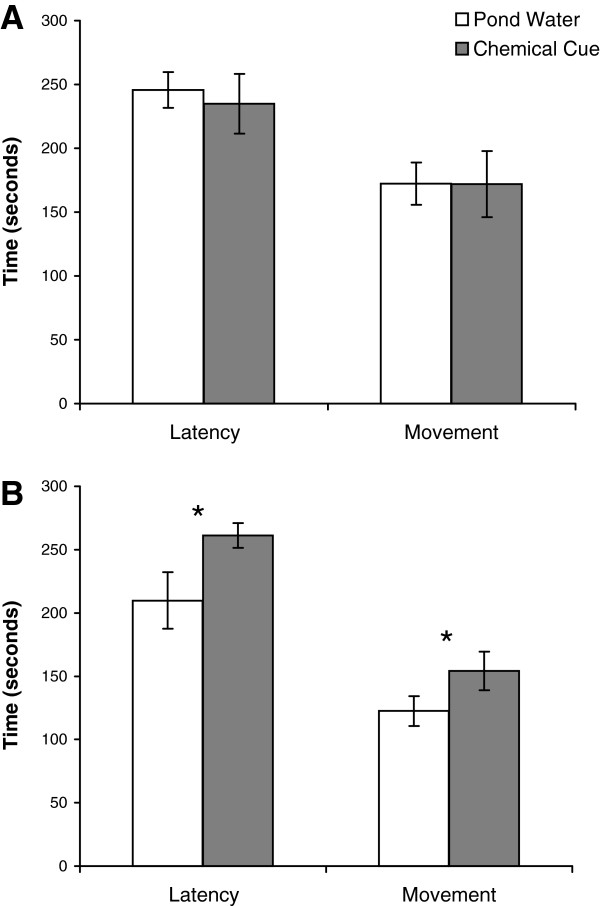
**Behavioral responses of female newts to chemical cues from a male-biased sex ratio.** Mean time spent per individual in latency and movement of females exposed to **A**) 2-male chemical cues or **B**) 4-male chemical cues compared to pond water used as a control. *N* = 8 females for each treatment. Means are ± 1 SE. * indicates *P* < 0.05.

We first measured the female behavioral response to a male-biased sex ratio using a repeated measures chemical cue test where the response to a male chemical cue was compared to the response to ambient pond water. Chemical cues play an important role in communication and identification of ambient sex ratio in red-spotted newts [35]. We compared the behavioral response of female newts when exposed to pond water versus either a two-male chemical cue or a four-male chemical cue. No difference was found in latency (*t* = 0.54, *P* = 0.60) or movement (*t* = 0.01, *P* = 0.99) between the two-male chemical cue treatment and pond water (Figure 4A). However, both latency (*t* = −2.39, *P* = 0.05) and movement (*t* = −3.99, *P* = 0.01) were significantly different between the four-male chemical cue treatment and pond water (Figure 4B). 

### Female newts spend more time in refuge habitat under a male-biased sex ratio

We conducted a second lab experiment to test our hypothesis that female newts adjust their microhabitat use in response to a male-biased sex ratio using aquariums with refuge habitat available. In general, we saw low amplexus rates in this experiment; males attempted courtship in only five aquaria in the female-biased treatment and one aquarium in the male-biased treatment during the 10-hour period. A chi-square test showed these discrepancies in amplexus frequency to be not significant (*χ*^2^ = 2.67, *P* = 0.102). For time not spent in amplexus, females in the male-biased treatment spent significantly more time in refuge than females in the female-biased treatment (mean proportional time in refuge = 0.45 for female-biased tanks and 0.70 for male-biased tanks; *F*_1,17_ = 6.67, *P* = 0.019).

## Discussion

Studies on sexual conflict have shown aggressive mating strategies employed by the common sex in biased populations often have profound impacts on the rare sex. These impacts can take the form of behavioral responses as well as direct physiological costs (reviewed in [37]). We measured the effect of male mating pressure on female condition in *N. viridescens* using replicated field experiments in natural habitat. In addition to a reduction in female body condition over the long-term, we found support for our hypothesis that sexual conflict leads to a reduction in female immune function. Through behavioral experiments, we found evidence that female newts may mitigate these negative impacts by altering their behavior and microhabitat use to avoid unwanted encounters with males.

In our long-term experiment we found that female body condition is negatively impacted by a male-biased environment, regardless of the density. In the low density enclosures resident females had a mean increase in mass in the female-biased treatment, but in the low density/male-biased treatment resident females lost just as much mass as in high density/male-biased enclosures (Figure 2). These results suggest that a biased sex ratio has a more dramatic impact on female body condition than competition through increased density. A portion of the mass loss experienced by females may be attributed to oviposition [29], but we expect the differences between treatments are largely due to increased resource competition under higher densities and a male-biased sex ratio. We expect females in the low density/female-biased treatment were able to acquire enough food resources over the long period of the experiment to compensate for oviposition. We also found that the male-biased treatment was costly to male newts. Negative consequences of increased male-male competition for males are common across animal species (reviewed in [1-3]) and expected in *N. viridescens* based on behavioral research showing that males increase energetically costly mating efforts under increasingly male-biased sex ratios [25,26]. In some species, density can influence or change male aggressive behavior [38]. However, we found the impact of the sex ratio treatment to be consistent across the density treatments, indicating that the male-biased environment, even at the low density, likely resulted in competitive mating behaviors as opposed to passive courtship [15,23].

In a species with partial migration, where a portion of the population remains as residents and the other portion migrates, females could be expected to adjust their migration timing or tactic to minimize time in the male-biased breeding pond. While a greater proportion of females migrate compared to males at the study site, both in this experiment and natural populations [21], manipulation of the sex ratio in the long-term experiment did not significantly affect migration timing or proportion migrating for either males or females [36]. Possibly the prolonged egg-laying season of *N. viridescens*, where females lay eggs singly over several weeks [29], prevents adjusting the departure timing from the pond. Alternatively, females may remain in the pond because it is superior habitat compared to the terrestrial forest in terms of survival and opportunities for feeding and growth [21,39]. Females may use the pond substrate and edge vegetation, which were not available inside the enclosures, both to successfully evade male newts and to maximize foraging time.

In our short-term field experiment we found that residing in a male-biased environment for five weeks had a negative effect on female immune function (decreased circulating leukocyte abundance), but resulted in no significant change in mass. Leukocyte profiling has been shown to reflect a physiological response to relatively immediate (i.e. within hours or days) stressors [32], which could explain why we saw a significant leukocyte response but no change in body mass. Also, the time period may have been too short to observe a significant change in mass as seen in the first experiment, due to the relatively low metabolic rate of ectothermic pond-breeding amphibians [40]; however hormonal indicators of proximate nutritive condition, such as leptin or glucocorticoids, may have been affected by this point [31]. The adaptive immune system has been shown to be highly sensitive to elevated glucocorticoids [41] or low leptin levels resulting from a reduction of energetic resources (reviewed in [31,42]), and this may explain the reduction in circulating leukocytes in females exposed to males in our study. Therefore, in this situation, immune activity may be a more sensitive indicator than body condition of physiological stress imposed by physical contact or by chemical/visual detection of males in the bins (also see [32]). Over the long-term, the combination of reduced body condition and suppression of adaptive immunity may result in significant fitness costs of a male-biased sex ratio on female newts, and future studies of the endocrine regulation of the adaptive immune system in this context would add to what is known about the ecological factors that affect immune function in amphibians (see [43,44]).

Our laboratory experiments show that female newts employ behavioral changes to minimize exposure to male mating harassment. The behavioral differences seen in the four-male chemical cue treatment were not seen in the two-male treatment, likely due to the concentration of pheromones being similar to ambient levels of chemical cues in natural pond water. This result is consistent with studies showing females newts to be averse to large groups of males and competitive mating encounters. In field trials, female *N. viridescens* avoided experimental groups containing four males [35]. In a European species of newt, *Triturus vulgaris*, females preferred single males to groups of males in a choice experiment [45].

Our habitat use hypothesis predicted that female newts avoid male harassment behavior by using different microhabitats within the pond environment. In our final behavioral experiment we found that females indeed spent significantly more time in refuge in a male-biased environment. We were unable to examine the role of refuge habitat on amplexus frequency due to low overall amplexus frequencies. To our knowledge, other studies on the mating behavior of this species have been conducted over continuous observation periods with individuals well acclimated to laboratory conditions [23,25]. Additionally, other studies of newt mating behavior have removed groupings from the study when amplexus is not observed after a short time period (e.g., [26]). The rate of amplexus in our experiment, observed in 7 of 30 aquaria, is similar to the rate observed by Gabor *et al*. [16], 11 matings in 30 trials, and may be within the normal mating rate for this species in a laboratory setting.

## Conclusions

This study illustrates the role of male harassment in the mating dynamics of an amphibian species. The red-spotted newt is an excellent example of a well-studied species where the effects of the mating system on females have been overlooked. We found support for our hypothesis that male mating harassment influences microhabitat use by females within the breeding pond and provides an explanation for differences between male and female newts in catchability. Female newts are averse to male-biased environments, which can incur costs to individual condition, and retreat into vegetation to avoid encounters with males. Examining the consequences of male mating behavior on female condition and behavior enhances our understanding of sexual conflict. Our study illustrates that the costs of male harassment can have significant impacts on female condition, immune function, and behavior, which ultimately have important consequences for the ecology of the species.

## Materials and methods

### Study site

All research took place at Mountain Lake Biological Station (Giles County, Virginia, USA, elevation 1160 m). The field experiments were conducted in Station Pond, a 0.65 hectare stream-fed, fishless permanent pond constructed in 1965. Station Pond contains a large population of *N. viridescens* (estimated newt population = 7,600–12,700 individuals; KL Grayson, unpublished data). The behavioral experiments were performed in a temperature and light controlled animal room in an aquatics laboratory on site.

### Long-term body condition experiment

Enclosures consisted of a large frame made from untreated pine lumber in the shape of a trapezoidal prism. The short end of the enclosure rested on the bank, leaving a small dry terrestrial area. Polyethylene netting on the sides and top (3.2 mm mesh, XV-1348, Industrial Netting, Minneapolis, MN, USA) was large enough to be permeable to small invertebrates, tadpoles and newt larvae but not adult newts. The bottoms of the enclosures were sealed with black plastic sheeting and 120 g of homogenized dry mixed oak leaf litter from the surrounding forest was added to each enclosure (see Appendix A of [36]).

Enclosures were placed around Station Pond in three spatial blocks on 14 April 2007. We used a 2 × 3 factorial design where we manipulated the population density (low = 12, medium = 24, high = 36 total newts) and sex ratio (2:1 or 1:2 males:females) within each enclosure. The male-biased sex ratio treatment was designed to reflect the estimated sex ratio of Station Pond (2.4–2.8 males: 1 female, KL Grayson, unpublished data). The female-biased sex ratio treatment was designed to reflect a release for female newts from male mating pressure. The treatments were assigned to enclosures using a randomized complete block design with unequal replication of the density treatments (replicates per sex ratio treatment: high density = 3, medium density = 6, and low density = 9 for a total of 36 enclosures with 720 newts). We increased the number of replicates for lower density treatments to increase the precision of estimates that were calculated from a lower number of individuals [36]. All newts used in the enclosures were collected from Station Pond, individually photographed, patted dry, weighed, and randomly assigned to enclosures on 23 April (males) and 25 April (females), before the majority of egg laying, which peaks in mid-June [29]. All newts used in the experiment displayed the aquatic phenotype [46], indicating their status as pond residents or fully transformed spring migrants into the pond.

Grayson & Wilbur [36] examined the effect of the treatments on migration outcome (staying as a pond resident overwinter or migrating to the terrestrial habitat). The terrestrial end of the enclosures was checked daily for newts attempting to migrate. Newts found out of the water could be confirmed as migratory due to a transition to a terrestrial phenotype (distinct dry, granular skin and reduced tail fin; [46]) and these individuals were removed, measured, and released in the surrounding forest. All remaining aquatic resident newts were collected on 15 September, photographed, patted dry, weighed, and released back into Station Pond. None of these individuals displayed characteristics of the terrestrial phenotype. The termination date was selected based migration patterns in studies of natural populations [18,21] and the very low number of migrants found after rains in September (92% of migrants found in this study were collected before 15 August). Photographs of the dorsal pattern of red spots were used to identify individuals (e.g., [18]). We used newts that remained in the enclosures until the end of the experiment (residents) to examine change in mass. Resident newts all spent the same amount of time in the enclosures, whereas migrants departed enclosures from June 1 – September 6.

We report body condition as the proportional change in body mass between the beginning of the experiment and the conclusion. When residuals from a regression of body mass and snout-vent length were used as the dependent variable, the same results were obtained. Change in body condition was analyzed using a generalized linear mixed model with a normal distribution of errors (PROC GLIMMIX, SAS). The three-way model included density treatment, sex ratio treatment, and newt sex as fixed effects and block as a random effect. In addition, to account for the common environment of individuals in the same enclosure, the variance among enclosures was used as the error term; this is tantamount to analyzing enclosure means. The data met normality assumptions without transformation. Newts from two enclosures were excluded from the analysis due to the low percentage of newts recovered [36].

### Short-term body condition experiment

Twenty-four 69 L plastic bins were modified by adding mesh openings (26 cm × 26 cm) to two sides of the rectangular bins (0.32 cm mesh as above). The top of each bin was covered with fiberglass screening secured with clothes pins to prevent newt escape. Bins were placed in the pond at least 60 cm apart at a depth of 27 cm and weighted with one large rock. We added 50 g of homogenized dry mixed oak leaf litter one week before newts were added. Each bin was randomly assigned a male-biased (2:1 males:females) or female-biased (1:2 males:females) treatment, with a total of 12 replicates in each treatment and a constant density of three newts per bin. Newts were collected from Station Pond and randomly assigned to bins, with one female per bin randomly designated as the focal female. Mass of focal females was recorded before the start of the experiment, in addition to number of dorsal red spots for later identification. Newts were added to the bins on 22 March and removed on 25 April 2009. Failure of the silicone caulk in a male-biased bin led to escape of an individual. This bin was excluded from all analyses.

Focal females were weighed after removal from the bins, euthanized by ventral Orajel® application [47], and then immediately decapitated. In all cases, three replicate blood smears of approximately 2-3μl of blood were prepared for each animal within 6 hours following removal from the field bins for immune function analysis. We attempted to collect blood through the tail without sacrificing, but found this method unsuccessful for these small newts.

To assess immune function, we measured circulating leukocyte and erythrocyte abundances from blood smears following the methods of previously published amphibian studies [43,44,48]. Blood smears were dried and fixed in a methanol solution then stained using Protocol Hema 3 Staining Kit (Fisher Cat. No. 122–911, Kalamazoo, MI, USA). Specific leukocyte types (lymphocytes, monocytes, eosinophils, basophils and neutrophils) were identified based on descriptions from Jain [49] and Wright [50]. We calculated leukocyte and erythrocyte density by haphazardly choosing 12 regions (170 μm × 230 μm each) of uniformly distributed cells with a compound light microscope (400x magnification); the total number of cells counted for each individual was approximately 2500–3200 total cells. Uniformity of cell counts among regions was assessed by comparing standard deviations of cell counts among regions of individuals, and cell regions were omitted and replaced if too large of a deviation was observed (e.g., due to clumping of cells or sparce cells at the end of the smear). A researcher blind to treatments counted cell types from digital images taken with a SPOT digital camera and imaging software (Diagnostic Instruments, Inc., Sterling Heights, MI, USA). Because of irregular or poor quality staining, one individual from the male-biased treatment was omitted from the analysis, leaving us with a sample size of n = 10 females from male-biased cages and n = 12 females from female-biased cages.

We compared the density of erythrocytes, total leukocytes, and lymphocytes (average cell counts/region), as well as the proportion of leukocytes to total number of cells, between females in male-biased or female-biased cages. Body mass was not significant as a covariate in any analysis, thus we used t-tests assuming equal variances to detect treatment effects on blood cell counts. We log transformed erythrocyte, total leukocyte and lymphocyte densities, and arcsin-square root transformed the proportion of leukocytes:total cell counts to normalize the data. Because the number of monocytes, neutropils, eosinophils, and basophiles were few and non-normally distributed, we summed the total number over all slide regions counted per individual and compared cell counts between treatments using a non-parametric signed rank test.

### Chemical cue response experiment

Male chemical cue was collected by placing either two males or four males in a plastic container (30 × 15.5 × 8.5 cm) filled with 1.5 L of pond water for one hour. Pond water containing ambient chemical signals from the natural sex ratio of Station Pond and was used to measure the baseline behavioral response of females.

Sixteen females were collected from Station Pond in July 2007 and eight were randomly assigned to receive chemical cue from two-males and the other eight females were assigned to receive chemical cue from four-males. The behavior of each female was recorded for four trials: two trials where only pond water was added and two trials where the assigned chemical cue water was added. The order of the trials was randomly determined. A female newt was placed in the same size plastic container as above filled with 1.5 L of pond water. A piece of plastic tubing was also placed in the container to add water after 10 minutes of acclimation time. A 50 ml syringe connected to the plastic tubing was used to add 30 ml of pond water or the assigned male chemical cue treatment water at the start of the trial. The duration of each trial was five minutes. Latency, the amount of time until the initial movement, and time spent in movement were recorded using an event recorder (Eve-Row 1990, J. Ha, Seattle, WA). A mean latency and movement response was calculated from the two chemical cue trials and the two pond water trials for each female. The difference in mean latency and movement between pond water and male chemical cue was tested using a paired t-test for females in the two-male and four-male chemical cue treatment.

### Refuge use experiment

Fifteen 38 liter aquariums were filled to a depth of 17 cm and we provided a refuge location in the center of the tank using a standard handful of saturated leaf litter from Station Pond. We ensured that refuge boundaries were clear and distinguishable from open areas of the tank by creating a formed pile of leaf litter. All aquaria contained three newts and were randomly assigned a sex ratio (1:2 males:females or 2:1 males:females). All newts used in the trials were captured the day before from Station Pond and housed in individual containers until the beginning of the observation period. We recorded newt behavior over a ten hour period from 7:30 to 17:30. Once per hour we recorded the behavior and position within the tank of each individual newt (either in open habitat, in refuge habitat, or in amplexus). A newt was considered in refuge habitat if more than 50% of its body was under leaf litter. The experiment was conducted on 5 April and 12 April 2009 for a total of 10 replicates in each treatment.

We compared proportional time spent in refuge between the 2:1 and 1:2 males:females treatments for females when not in amplexus (as females could only make a habitat decision when not grasped by a male). For the female-biased treatment we used the mean proportional refuge time for the two females in each aquarium. We used a linear mixed model (PROC MIXED, SAS) to compare proportional refuge time between the two treatments. We included date of the trial as a random effect and arcsine square-root transformed the dependent variable. All statistics were conducted in JMP 8 or SAS 9.1 (SAS Institute, Cary, NC, USA).

## Competing interests

The authors declare that they have no competing interests.

## Authors’ contributions

KLG conceived of the study, designed and executed the long-term field experiment, and contributed to data analysis and writing of the manuscript, SPD designed and executed the short-term field experiment and the refuge use experiment and contributed to data analysis and writing of the manuscript, JEJ executed the chemical cue response experiment, EJC and SJB collected data from the blood smears, and EJC assisted with the design of the short-term field experiment, analyzed the data from the blood smears, and contributed to the writing of the manuscript. All authors read and approved the final manuscript.
